# Hair Transplantation Surgery - Its Current Status

**DOI:** 10.4103/0974-2077.69013

**Published:** 2010

**Authors:** Venkataram Mysore

**Affiliations:** *Venkat Charmalaya - Centre for Advanced Dermatology, Bangalore, Karnataka, India*

This edition of the journal focuses on hair restoration surgery. The field has seen major advances in recent years and has rightfully caught the imagination of patients and doctors alike, and hence, the focus on this topic. This issue carries articles on factors affecting graft survival, prevention of postoperative oedema, follicular unit extraction method of transplantation, possible link between finasteride and male breast carcinoma and role of alternative regimes in medical management.

Several advances have lead to this newfound popularity of the technique; identification of the follicular unit[1] and elucidation of its role in both follicular unit transplantation and extraction methods, new methods of donor strip dissection such as trichophytic closure[2] and improved techniques in donor dissection using stereomicroscopes.[3] Enhanced training techniques and improved team work have resulted in larger sessions being performed in lesser time than before.[4] Publicity in media and the Internet have also played their role in creating awareness among patients. Scores of websites and blogs now discuss threadbare every aspect of this technique, with prospective patients and post-surgery patients driving the discussions.

The number of transplants being performed have, as a result, increased worldwide. In India too, this technique has arrived! While there were only five members of International society of hair restoration surgery in 2002, there were as many as 150 members in the Association of hair restoration surgeons (India) within the first year of its founding. The first conference of the association held in Ahmedabad in 2009 saw as many as 150 delegates. Fellowships for training are available in India and Indian hair restoration surgeons are making significant contributions to the field. There is a heightened interest about the procedure in the medical profession, and many young doctors are keen to learn it.

Have the advances in the field lead to enhanced patient satisfaction? Certainly, results produced with current techniques are impressive, and with megasessions of over 3000 grafts (even 6000 grafts in the hands of some surgeons), most, if not all, cases of baldness can be treated in a single session. Trichophytic closure and greater awareness of factors that affect scar formation have improved our closure methods and resulted in scars, which are better than ever before. Recipient density too has improved, with surgeons achieving more and more density than ever before (whether this is desirable in view of the limited donor that is available is an entirely different question). We also have newer methods to enhance survival of grafts, as discussed in lucid detail by Parsely WM and Perez-Meza D in their article in this issue.

Despite these advances, patients ask for more, long for more and the debate on most blog sites reflects this undercurrent dissatisfaction. This is partly fuelled by the ever increasing thirst for better results, and due to the changing social trends and attitudes. This is further enhanced by the open debates taking place in the Internet blogs and also the tendency of some surgeons to exaggerate their results or techniques.

Two such techniques reflect these aspects- Follicular unit extraction (FUE) and Body hair transplantation (BHT). Despite the recent advances, such as trichophytic closure, not every one gets a pencil line scar after strip method, which would be visible if the hairs were kept short. FUE discussed in this issue by Dua A and Dua K, emerged as a result of such a trend to keep hairs short and the dissatisfaction with the linear scar of strip method. The rationale for the method was first provided by Rassman[5] in their fascinating article. Derided initially as a reinvention of the disgraced punch, it has now established its place in hair restoration, with nearly 10% of all transplants being done by this method.[6] There has been an explosion of Internet sites eulogizing and even exaggerating the advantages of this method and deprecating the strip. However, despite it being an exciting advance, it is less perfect than FUT. It is slower, results in more transection of follicles, is more expensive and therefore presently suited for small areas. However, advances have been taking place, with newer methods of automation being introduced, which should result in faster and more accurate extraction and perhaps lessening of costs. Surgeons are already performing megasessions of up to 2000 grafts per day. Certainly, FUE wound heals faster and gives freedom to the patient to keep the hairs short. Most importantly it can be combined with FUT to increase the number of grafts given to a patient, without taking wider strips.

Body hair, originally described by Woods R has enhanced the available source of grafts.[7] The article by Hwang S,[8] for which he received the platinum follicle award by the International society for hair restoration surgery(ISHRS), established the concept of recipient influence and showed that body hairs can grow longer when transplanted to scalp. The technique of FUE is eminently suited to extract body hair and has enabled us to give an ever greater number of grafts to a patient over 2–3 days. However, like FUE, it is slow, expensive and awaits techniques to improve the rate and precision of extraction. However, as in the case of FUE, exaggerated projection of the technique on Internet sites have lead to many patients seeking the procedure without being aware of the disadvantages.

Obviously, what is needed is a balanced evaluation of the procedures, and proper counselling of patients. While these significant advances have been taking place on the surgical front, it is some what depressing that advances in preservation of existing hair been slow. We still have only two approved drugs; minoxidil and finasteride. The limitations of the former such as irritability, difficulty in application and waning of effect after 3–4 years are well known. Patient compliance is therefore a major problem. Foam delivery has some what improved patient compliance, and one hopes liposomal delivery will bring more cheer to patients. Finasteride continues to receive bad publicity among patients regarding its role in impotence, whatever be the scientific evidence to the contrary. Recent reports about the possible occurrence of male breast carcinoma in patients receiving finasteride are somewhat disturbing. Drs Shenoy N and Sangolli P discuss this topic in this issue and have summarized a recent report of Medicines and Health care products Regulatory Agency (MHRA). It is, therefore, obvious that unless there is a satisfactory medical therapy to control the progression of baldness, any advances in transplantation would only result in temporary improvement for the patient. This would need further advances in our knowledge of the mechanisms and the causes of baldness. It is well known that not all patients of pattern hairloss respond to finasteride, suggesting the possible role of alternative factors. An article by Rajput R explores the alternative hypothesis for pattern hairloss in this issue and looks in to somewhat controversial issue of the role of nutritional factors, antioxidants etc.

The field of hair restoration is perhaps in its golden period; hair restoration surgeons have never had it so good. It is important for the practitioners of the field to emphasize standards of care, proper training of new entrants in to the field and ensure ethical conduct in advertisements and patient information. A recent publication by a taskforce of dermatosurgery of Indian association of dermatologists, leprologists and venereologists has laid down such standards of care in hair transplantation.[9] A cogent article by Goh CL[10] published in an earlier issue of this journal, emphasized the need for evidence based aesthetic practice, proper documentation and ethical evaluation of new therapies before their adaptation in practice. Such practices would not only ensure proper health delivery, but also enforce the ethical standards required to maintain the image of this rapidly progressing field.
